# Do bulls experience pain or stress during electroejaculation? Evidence from electroencephalography, behavioral, hormonal, and metabolite profiling

**DOI:** 10.14202/vetworld.2025.763-772

**Published:** 2025-04-07

**Authors:** Ubedullah Kaka, Nurhusien Yimer Degu, Pavan Kumar, Abubakar Ahmed Abubakar, Yong-Meng Goh, Muhammad Waseem Aslam, Khaleeq Ur Rehman Bhutto, Muhammad Abdul Basit, Wasim S. M. Qadi, Norazlan Mohmad Misnan, Ahmed Mediani, Khor Kuan Hua

**Affiliations:** 1 Department of Companion Animal Medicine and Surgery, Faculty of Veterinary Medicine, Universiti Putra Malaysia, 43400, Selangor, Malaysia; 2 Halal Products Research Institute, Putra Infoport, Universiti Putra Malaysia, 43400, Selangor, Malaysia; 3 Department of Veterinary Sciences, School of Medicine, IMU University, Bukit Jalil, 57000, Kuala Lumpur, Malaysia; 4 Division of Veterinary Reproduction, Faculty of Veterinary Medicine, Universitas Airlangga, 60115, Surabaya, Indonesia; 5 Institute of Tropical Agriculture and Food Security, Universiti Putra Malaysia, 43400 UPM Serdang, Selangor, Malaysia; 6 Department of Livestock Products Technology, College of Veterinary Science, Guru Angad Dev Veterinary and Animal Sciences University, Ludhiana, Punjab, India; 7 Department of Preclinical Sciences, Faculty of Veterinary Medicine, Universiti Putra Malaysia, 43400, Selangor, Malaysia; 8 Department of Clinical Studies, Faculty of Veterinary Medicine, Universiti Putra Malaysia, 43400, Selangor, Malaysia; 9 Department of Biosciences, Faculty of Veterinary Sciences, Bahauddin Zakariya University, Multan, 60000, Punjab, Pakistan; 10 Institute of Systems Biology (INBIOSIS), Universiti Kebangsaan Malaysia, Bangi, Selangor 43600, Malaysia.; 11 Herbal Medicine Research Centre, Institute for Medical Research, National Institutes of Health, Shah Alam, 40170, Malaysia

**Keywords:** bulls, electroejaculation, electroencephalography, hormonal indicators, metabolomics, pain, stress

## Abstract

**Background and Aim:**

Electroejaculation (EE) is widely used for semen collection in bulls but raises concerns about animal welfare due to potential pain and stress. The physiological impact of EE on bulls remains a topic of debate, with previous studies yielding inconclusive results. This study aims to objectively evaluate pain and stress responses in bulls subjected to EE using electroencephalography (EEG) alongside hormonal, behavioral, and metabolite profiling.

**Materials and Methods:**

Eight bulls were subjected to EE in three replicates, with physiological and behavioral data collected before, during, and after the procedure. EEG parameters, including median frequency (MF) and total power (Ptot), were analyzed to assess cortical activity indicative of pain and stress. Blood samples were evaluated for stress-related hormones (adrenaline, noradrenaline, β-endorphin, and dopamine), while metabolomic analysis was conducted to identify biochemical alterations associated with stress. Behavioral indicators, including vocalization and muscle spasms, were recorded.

**Results:**

EE induced significant increases (p < 0.05) in stress hormones at ejaculation, which gradually returned to baseline 20 min post-procedure. EEG metrics, such as MF and Ptot, significantly increased during EE (p < 0.05), indicating heightened cortical activity associated with nociception. Metabolomic analysis revealed distinct biochemical shifts, with variations in glucose, taurine, and norepinephrine profiles across baseline, stimulation, and recovery phases. Behavioral observations corroborated physiological findings, with bulls exhibiting signs of discomfort, such as struggling, arched back posture, and excessive salivation.

**Conclusion:**

The combined EEG, hormonal, and metabolomic findings confirm that EE is a stressful and painful procedure for bulls. The study provides robust evidence of neurophysiological and biochemical responses indicative of pain. These findings highlight the need for alternative semen collection methods to minimize animal distress and improve welfare standards.

## INTRODUCTION

Semen from bulls can be collected using different methods; through the artificial vagina, transrectal massage, and electroejaculation (EE). EE enables the collection of semen from sexually mature animals for the evaluation of breeding soundness and artificial insemination. The procedure involves stimulation of the pelvic nerves and surrounding tissues with an electrical current. Stimulus duration, rest intervals, and voltage increments can influence the level of pain response. The correct placement and orientation of the probe are important for minimizing the pain response to the EE [1]. Due to welfare concerns, the European Union has banned the importation of frozen semen collected through EE. However, this method is still employed in several countries, such as Malaysia, India, and Pakistan. EE is considered a painful procedure and is believed to cause severe stress in animals, that warrants further investigation.

When the central nervous system perceives a stress or painful stimulus as a threat, it activates the biological defense system, including the behavioral, autonomic nervous system (ANS), neuroendocrine, and immune responses [2]. Conventionally, pain and stress are measured through behavior, endocrine system, and the immune system [3, 4]. However, there are some drawbacks to using endocrine and immune systems as indicators of welfare status and stress biomarkers. For example, a single sample collected at a specific time may not correspond to the level of pain that the animal is experiencing. Similarly, alterations in the plasma levels of hormones indicating pain could manifest as other stress responses. On the one hand, these biochemical- and hormonal-based methods often have a lag time following a stress-induced change. On the other hand, ANS, which involves action potential trans- mission through neurons in the brain, is the instanta-neous and fastest response among all changes initiated in response to stress stimuli. Electroencephalography (EEG) is an objective, noninvasive, and stress-free technique to record instantaneous physiological responses to both stress and nociception (pain) in animals. EEG is a real-time graphical representation of tiny (of the microvolt range) spontaneously generated electrical currents of neurons from the cerebral cortex through electrodes placed on various positions on the scalp in humans or heads in other species [5]. EEG has been used to measure pain in response to noxious (painful) stimuli in animals [4, 6]. There is a dearth of literature regarding the pain experienced during EE in bulls, and it is the subject of debate from animal welfare perspectives. A previous study by Whitlock *et al*. [3] evaluated the behavioral reaction, such as vocalization and neuropeptides, such as substance P (unaffected), in response to EE in bulls.

Metabolomics is closely linked to the metabolome, which comprises small molecules within cells. Studying and assessing changes in these small molecules under different conditions is a powerful method for understanding biological systems. As a global characterization tool for identifying metabolites, metabolomics has become crucial in biological regulation, including disease prevention and treatment [7]. Recent technological advances have propelled metabolomics into a phase of rapid growth in biology and biomedicine. Discoveries of metabolites and their mechanisms provide a solid foundation for further research. One key technique in this field is proton nuclear magnetic resonance (1H NMR), which is gaining popularity due to its ability to identify various compounds. Using 1H NMR-based metabolomics, researchers can evaluate changes in biofluid composition, animal behaviors, and physiological changes. Thus, it is a powerful tool for assessing metabolome variations [8, 9].

Despite the widespread use of EE for semen collection in bulls, there is a limited understanding of the precise neurophysiological and metabolic stress responses associated with this procedure. While previous studies have focused on behavioral and hormonal indicators of stress, there is a lack of objective, real-time assessments of pain perception using EEG and metabolomics. Moreover, the existing literature does not comprehensively elucidate the biochemical pathways influenced by EE, leaving a critical gap in knowledge regarding the physiological impact of this technique on animal welfare.

This study aims to assess the pain and stress responses in bulls subjected to EE by integrating EEG analysis with hormonal, behavioral, and metabolomic profiling, thereby providing a comprehensive and objective evaluation of the physiological impact of this procedure on animal welfare.

## MATERIALS AND METHODS

### Ethical approval

This study was approved by the Universiti Putra Malaysia Animal Care and Use Committee (UPM/IACUC/AUP-U031/2020) and conformed to the Malaysian Code of Practice in the Care and Use of Animals for Scientific Purposes.

### Study period and location

This study was conducted during August and September 2020, at Ladang Taman Pertanian University (TPU), Universiti Putra Malaysia

### Experimental design

A total of 8 bulls (Kedah-Kelantan) and Friesian cross bulls (each with an average age of 2 years) in 3 replicates (sample size − 8 bulls × 3 replicate = 24) were used in this study. The bulls were kept at Taman Pertanian Universiti, Universiti Putra Malaysia, during the study period. These bulls were fed *ad libitum* with *Brachia decumbens* grass and palm kernel cake. The bulls were derived from the pen to the chute one by one and were appropriately restrained with minimal stress before conducting the procedure [10]. It is worth mentioning that the bulls were trained in the same chute and were subjected to this procedure routinely before the experiments.

On the day of the experiment, one bull was selec- ted randomly and derived gently into the chute, where the attending technician interacted with the bull. EEG was recorded using two adhesive electrodes (as described in the EEG recording section), and EEG was recorded for 2–5 min, followed by blood sample collection (baseline). After collecting the baseline data and samples, the animal was prepared for semen collection by clearing feces from the rectum and inserting an electrical probe. EEG data were recorded throughout the EE process until ejaculation. Blood samples were collected around the time of ejaculation. After the completion of the EE procedure, the bull was allowed to rest for 20 min, followed by blood sampling and EEG recording for 2 min (post-ejaculation).

### Preparation of bulls for semen collection

#### The operation of the electroejaculator

An electro-ejaculator machine (ElectroJac 6; Neogen®Corporation, Lansing, IM48912; L24290812) was used to stimulate the bulls and collect semen. During the procedure, a rectal probe (51 mm in dia-meter and 330 mm in length) with three longitudinally arranged electrodes, lubricated and connected to the EE machine, was inserted into the rectum. While one person held the probe in position inside the rectum, the other operated and monitored the power output cycles from the EE machine and the behavioral response of the bulls. A modified auto-manual combined operational protocol (semi-automatic protocol) routinely used in the farm was employed to stimulate the bull for ejaculation. Before the start, the battery switch was kept in the “ON” mode manually, and the EE was set in “ON” by pressing the green button on the remote control, with the Auto/Manual knob set at a low level. Initial stimulation was then provided by turning the knob anti-clockwise to the auto-position, during which the power output progression cycle was allowed to rise to 3–4 cycles and then returned to zero. This step was repeated 2–3 times before the power output cycle was allowed to progress to a higher level, leading to erection and semen ejaculation by the bull. The bulls ejaculated semen within a range of 6–12 power output cycles. Each cycle is characterized by 2 s “ON” and 2 s “OFF,” a pause in the power output gradually increasing with a slight voltage intensity.

### Blood samples

Ten milliliters of blood was collected using an 18-gauge needle through jugular venipuncture with an aseptic technique into vacutainer K3 ethylenediaminetetraacetic acid tubes (BD Franklin Lakes, NJ, USA). The blood samples were collected into a vacutainer, slanted in crushed ice, and taken to the Clinical Pathology and Hematology Laboratory, Faculty of Veterinary Medicine, Universiti Putra Malaysia, after approximately 1 h of collection. The samples were centrifuged at 800 rpm, 4°C for 15 min. The retrieved plasma portion was separated into 2 mL aliquots and kept at –80°C until the analysis for adrenaline, nor-adrenaline, β-endorphin, and dopamine.

Blood plasma adrenaline and nor-adrenaline concentrations were analyzed quantitatively using commercially available adrenaline and nor-adrenaline plasma enzyme-linked immunosorbent assay (ELISA) kits (Qayee-Biotechnology Co. Ltd, Shanghai, China), respectively. The β-endorphin and dopamine were determined using commercially available ELISA kits specifically for bovine (Qayee-Biotechnology Co. Ltd) following the manufacturer’s instructions.

### Metabolomics analysis of serum samples

The serum was separated after collecting the blood. The serum was maintained at –80°C before analysis with nuclear magnetic resonance (NMR). The NMR preparation and analysis methods were carried out in accordance with a previously reported protocol [11]. The serum samples were thawed before centrifugation at 1200× *g* for 5 min. Subsequently, 400 μL of KH_2_PO_4_ buffer in D_2_O (pH 7.4) containing 0.2% trimethylsilylpropanoic acid (TSP) was added to 200 uL of serum supernatant in Eppendorf tubes. The mixtures were vortexed and centrifuged again for 10 min at 3000× *g*. The supernatant was then transferred in 550 μL to an NMR tube. NMR spectra metabolite profiles from the samples were obtained on a JNM-ECZ-600R 600 MHz spectrometer (JEOL, Tokyo, Japan). Water suppression analysis using Carr–Purcell–Meiboom–Gill pulse sequences was used to suppress large residual water resonance or broad protein resonance and to enable the signals of small molecules to be portrayed. This analysis was performed using the following parameters: Spectra width of 15 parts per million (ppm), acquisition time of 3 s, relaxation delay time of 7.0 s, 90° pulse width, temperature of 293 K, and 64 scans. One-dimensional 1H NMR experiments of the sample were performed using a single pulse sequence. The D_2_O chemical shift peak was suppressed and used as an internal reference. The parameters used in this analysis were a spectral width of 20 ppm, time domain data points 16 K, flip angle 45, relaxation delay 5 s, spectrum size 16 K, and number of scans 32. The spectra were manually phased using the JEOL Delta processing software (v5.1.3) (JEOL Ltd, Tokyo, Japan). The baseline was then corrected and referenced to TSP (δ 0.00). Furthermore, two-dimensional (2D) J-resolved NMR was used to resolve overlapping metabolite signals. The library of Chenomx NMR Suite (v. 8.2, Alberta, Canada), the free online Human Metabolome Database (University of Alberta, Canada), and previously reported data were used for metabolite identification. The typical metabolites for each group were determined through multivariate data analysis (MVDA).

### Behavioral response

Two staff members trained in the behavioral assessment of cattle recorded the behavior of the bulls, such as vocalization, arched back, intense muscle spasm (contraction of muscles in thighs and abdominal region during semen collection using EE), and struggling (intense movements of hind limbs, including kicking, trying to move backward and forward during EE and salivation), through close observations [3, 10].

### EEG recording

EEG activity was recorded using a power laboratory recording system (Power Lab data acquisition system, AD Instruments Ltd. Sydney, Australia). Two Kendall™ (Covidien 11c, 15 Hampshire Street, Mansfield 02048 USA) conductive adhesive hydrogel foam electrodes were used; the inverting (negative electrode) was attached 6–8 cm distally from the poll, equidistant to both the anterior orbital prominences of the left and right eyes. A second non-inverting (positive electrode) was attached to the mastoid process. Before recording, the area was shaved and cleaned with 70% alcohol. The EEG signals were recorded at a sampling rate of 1 kHz, and raw EEG was re-sampled with a low-pass filter of 200 Hz into delta frequency (0.1–4 Hz), theta frequency (4.1–8 Hz), alpha frequency (8.1–12 Hz), and beta frequency (12.1–20 Hz). The EEG data analysis was performed offline after the completion of experiments using the Chart Spectral Analysis Function (Chart 5.0TM software) (Powerlab^TM^ data acquisition system, Sydney, Australia).

Before analysis, possible interferences from concurrent electrocardiograph signals were digitally removed from the raw EEG recordings using the Chart 5.0 TM software. Signals were then processed for consecutive non-overlapping 1-s epochs, yielding 60 epochs per minute. The root mean square for each alpha, beta, delta, and theta wave was calculated before and after EE. The median frequency (MF) is the frequency below which 50% of the total power (Ptot) of the EEG is located. The Ptot, which is the total area under the curve, was also determined.

### Statistical analysis

Statistical analysis was conducted using SAS software version 9.4 (SAS Institute Inc., Cary, NC, USA). The data were first tested for normality using the Shapiro–Wilk test. Comparisons between baseline, during EE, and 20 min post-EE were performed using Duncan’s multiple range test. p < 0.05 was considered statistically significant.

For metabolomic data analysis, nuclear magnetic resonance (NMR) spectral binning was performed at intervals of 0.04 ppm, and the spectra were normalized using the TSP signal. Water signals (δ 4.60–4.85 ppm) were excluded from the dataset. MVDA was conducted using Simca-P software (version 14, Umeå, Sweden) to identify variations among experimental groups. Principal component analysis (PCA) was used to visualize sample clustering, while partial least squares discriminant analysis (PLS-DA) was applied to further assess metabolite variations between groups.

In addition, a heatmap was generated to illustrate differences in metabolite concentrations across baseline, post-stimulation, and recovery phases. Variable Importance in Projection (VIP) scores from PLS-DA were used to determine the most significant metabolites contributing to group differentiation. The pathway impact analysis was performed using MetaboAnalyst 5.0 (http://www.metaboanalyst.com), incorporating data from the METLIN (La Jolla, California, USA) and Kyoto Encyclopedia of Genes and Genomes (KEGG) databases (http://www.genome.ad.jp/kegg/) (Kyoto, Japan). Model validation was assessed using regression values (R²) and permutation tests to confirm the robustness of the statistical analysis.

## RESULTS AND DISCUSSION

### Hormonal responses

The EE of bulls was observed to have a significant (p < 0.0001) increase in the hormonal responses (Table 1). The highest plasma concentrations of catecholamines, beta-endorphin, and dopamine were recorded during EE and decreased 20 min post-ejaculation. The 20 min post-ejaculation catecholamine concentration was comparable to the baseline values. In contrast, plasma β-endorphin and dopamine concentrations were still higher than baseline values (β-endorphin 149.507 ± 2.27 vs. 139.824 ± 2.01 and dopamine 24.765 ± 0.72 vs. 20.926 ± 1.02) 20 min post-ejaculation. However, they were significantly lower than the values during EE (β-endorphin 170.534 ± 1.51 vs. 149.507 ± 2.27 and dopamine 28.416 ± 0.69 vs 24.765 ± 0.72) (Table 1).

**Table 1:** Hormonal responses to EE in bulls.

Parameters	Baseline	Ejaculation	Relax*	p-value
Adrenaline (pg/mL)	1.27 ± 0.02^b^	1.79 ± 0.09^a^	1.39 ± 0.09^b^	<0.0001
Noradrenaline (pg/mL)	21.18 ± 0.44^b^	27.72 ± 0.56^a^	22.58 ± 0.74^b^	<0.0001
β-endorphin (pg/mL)	139.824 ± 2.01^c^	170.534 ± 1.51^a^	149.507 ± 2.27^b^	<0.0001
Dopamine (pg/mL)	20.926 ± 1.02^c^	28.416 ± 0.69^a^	24.765 ± 0.72^b^	<0.0001

Mean ± SE, n = 24;

* 20 min post-electroejaculation. EE=Electroejaculation, SE=Standard error

Assessment of hormones such as catecholamines and β-endorphins gives deep insight into the animal welfare, such as stress or painful conditions [12, 13]. The rapid influx of plasma catecholamines in the present study may be due to electrical stimulation of pelvic nerves and surrounding tissues. This electrical stimulation could act as a stressor in bulls, thereby leading to the activation of the sympathoadrenal system (SPA) and the release of catecholamines by the adrenal medulla. Increased plasma β-endorphin indicates stressful conditions rather than pain [14, 15]. Increased plasma β-endorphin levels in this study indicate stress and pain associated with EE in bulls. Similarly, increased plasma β-endorphin levels were reported under various stressful or painful procedures in livestock, such as tail docking, restraints, and castration in lambs [16], labor pain in goats [17], and acute and chronic pain in rats [18, 19]. Interestingly, plasma dopamine concentrations increased during the EE phase in bulls.

Dopamine is a neurotransmitter produced in the brain under pleasurable and motivational conditions. Dopamine plays a significant role in facilitating the effects of copulatory efficiency, sexual behavior, and genital reflexes [20]. The significantly higher dopamine levels at the time of semen ejaculation could be attributed to the pleasure (although painful) stage, as ejaculation per se is not a painful phenomenon [1]. Nervous stimulation and muscular damage in response to electric stimulation may have caused stress and painful responses in bulls in this study. A higher plasma cortisol concentration and creatine kinase levels were reported due to EE in deers [21], rams [22], and bucks [23], thereby indicating stress and tissue damage.

Neuroendocrine is one of the four biological defense mechanisms in response to stress or painful stimulus, perceived as a threat by the central nervous system, leading to the release of catecholamines due to activation of SPA and release of cortisol by hypothalamic-pituitary-adrenal (HPA) in persistent stress/threat [2]. Thus, an increase in adrenaline, noradrenaline, β-endorphin, and dopamine levels in response to EE and their return to baseline after 20 min indicates that EE is stressful and painful for bulls.

### EEG variables

Table 2 shows the EEG variables in response to EE in bulls (Table 2). The MF increased significantly (p ≤ 0.0001) from 13.92 ± 8.02 Hz at baseline to 38.25 ± 18.66 Hz at ejaculation and returned to baseline values of 13. 55 ± 7.67 Hz 20 min post-ejaculation. A similar trend was observed for Ptot, which increased significantly (p ≤ 0.0001) from 8.55 ± 2.34 μV at baseline to 29.83 ± 14.56 μV at ejaculation; the values returned toward the baseline of 8.23 ± 2.52 μV, 20 min post-ejaculation. Likewise, alpha, beta, delta, and theta increased significantly (p ≤ 0.0001) at EE compared with baseline and returned to baseline values 20 min post-ejaculation.

**Table 2:** Electroencephalogram responses to EE in bulls.

Parameters	Baseline	Ejaculation	Relax*	p-value
Alpha (µV)	1.14 ± 0.04^b^	2.38 ± 0.13^a^	1.28 ± 0.05^b^	<0.0001
Beta (µV)	1.95 ± 0.05^b^	4.38 ± 0.22^a^	2.16 ± 0.06^b^	<0.0001
Delta (µV)	5.21 ± 0.18^b^	16.19 ± 1.09^a^	4.97 ± 0.17^b^	<0.0001
Theta (µV)	1.39 ± 0.05^b^	2.82 ± 0.19^a^	1.49 ± 0.06^b^	<0.0001
Ptot (V^2^/Hz)	8.69 ± 1.20^b^	29.75 ± 1.20^a^	8.33 ± 0.19^b^	<0.0001
MF (Hz)	13.04 ± 0.68^b^	39.55 ± 1.56^a^	14.95 ± 0.61^b^	<0.0001

Mean ± SE, n = 24 20 min post-electroejaculation. MF=Median frequency, EE=Electroejaculation, SE=Standard error

Through measurement of the electrical activity of cerebrocortical neurons, EEG has proven to be a real-time, sensitive, and reliable indicator of brain activity under particular conditions, such as pain and stress, in animals [6, 13, 24]. Analysis of the EEG spectrum has been used to assess the pain and stress in ruminants [25, 26]. The increase in the EEG power spectrum observed in this study could be well correlated with the increased plasma catecholamine concentration.

Increased handling of animals and environmental changes are also associated with higher alpha-wave activity. The increase in alpha wave activity during transportation stress was recorded in goats [27, 28]. Increased beta wave activity in humans indicates stressful conditions [29]. Higher beta wave activity was observed in panic conditions and fear perceptions [30]. Higher delta wave activity indicates arousal and alertness [31]. Thus, the increase in EEG wave activity in bulls during EE in the present study may indicate that bulls were in a state of alertness rather than relaxed state.

In the present study, Ptot and MF increased by 3.4- and 3-times during EE, respectively, compared with baseline values. The MF and ptot of the EEG were reported to be very reliable indicators of pain and stress in previous studies on pain and stress in animals [6, 13, 24, 26]. The Ptot value of the EEG spectrum was also observed to increase during stressful conditions in animals [32, 33]. The MF of the EEG spectrum is also correlated with stress and pain in goats [28]. An increase (p < 0.01) in Ptot and MF of the EEG spectrum was reported due to stress and pain caused during castration in rams [34]. Thus, in line with previous studies, the increase in MF and ptot from baseline and return toward the baseline after 20 min indicates that bulls were in stress and pain during EE in this study.

### Metabolite changes

**Figure 1:**
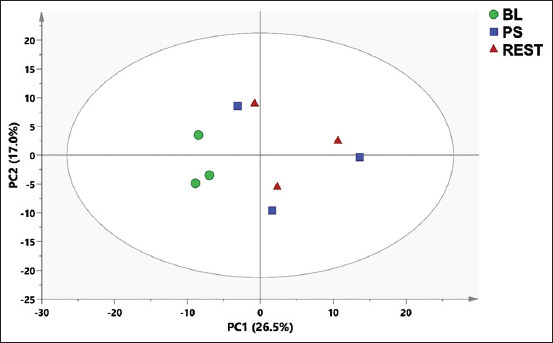
Principal component analysis score plot of sample name (BL, PS, and REST). Partial least squares discriminant analysis. BL=Baseline, PS=Post stimulus and REST=Resting.

**Figure 2:**
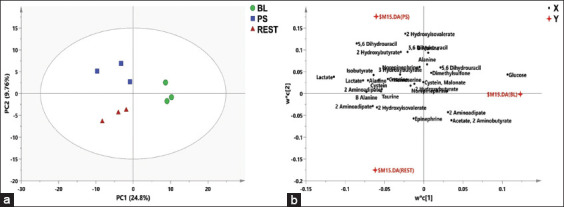
(a) PLS-DA score plot of sample name (BL, PS and REST). (b) PLS-DA loading plot of sample name (BL, PS, and REST. PLS-DA=Partial least squares discriminant analysis, BL=Baseline, PS=Post stimulus, and REST=Resting.

PCA was applied to determine the clustering features of the samples and the metabolites that contributed to the variability. The PCA score plot (Figure 1) showed no sample clustering. PLS-DA was applied to determine the variation among the samples. The variation among the metabolites in the baseline (BL), post-stimulus (PS), and resting (REST) was performed using a PLS-DA score plot (Figure 2a), while a loading plot (Figure 2b) was applied to identify the metabolites that contributed to the discrimination among samples in PLS-DA score plots. According to the PLS-DA score plot, the first principal component (PC1) could explain 24.8% of the total variation in the data. In contrast, PC2 only accounted for 9.76% of the total variation. The score plot shows three clusters: BL, PS, and REST. The loading plot (Figure 2b) clarifies the metabolites responsible for this variation between samples. The metabolites, glucose, 2 amino butyrate, cysteine, acetate, 5.6 dihydrouracil, dimethyl sulfone, malonate, and alanine were prevalent in BL. However, epinephrine, taurine, β alanine, 2 hydroxy isovalerate, and 2 aminoadipate were prevalent in REST. In addition, norepinephrine, creatine, 5.6 dihydrouracil, 2 hydroxybutyrate, isobutyrate, lactate, and serine were prevalent in PS.

**Figure 3:**
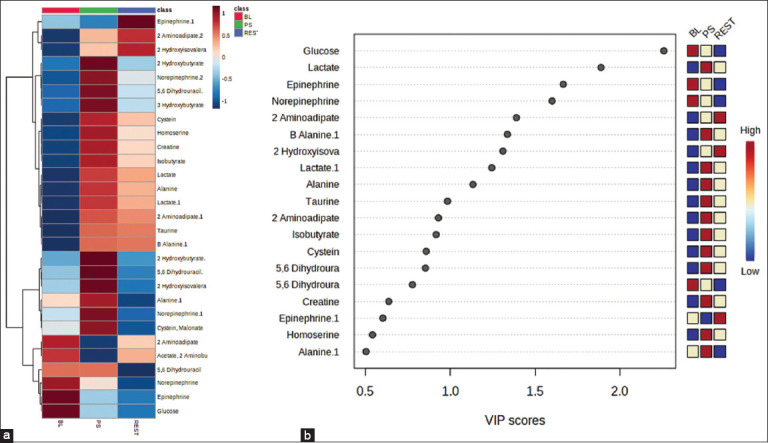
(a) Heatmap showing differences in metabolites of BL, PS, and REST. The color of the squares is associated with metabolite differences. (b) The variables are essential in the projection values derived from partial least squares, showing significant metabolite differences among groups. The dark red color has the most substantial contribution, whereas dark blue has the least contribution. BL=Baseline, PS=Post stimulus and REST=Resting.

The heatmap was applied to show the metabolite concentration differences among the BL, PS, and REST (Figure 3a), which supports the PLS-DA loading plot result. The VIP values were applied using the PLS-derived biplots to determine the main factors contributing to the metabolite’s variation among the samples (Figure 3b). The VIP values (>0.5) are significant in the projection in the PLS model (Figure 3a).

### Metabolic pathway analysis of EE

**Figure 4:**
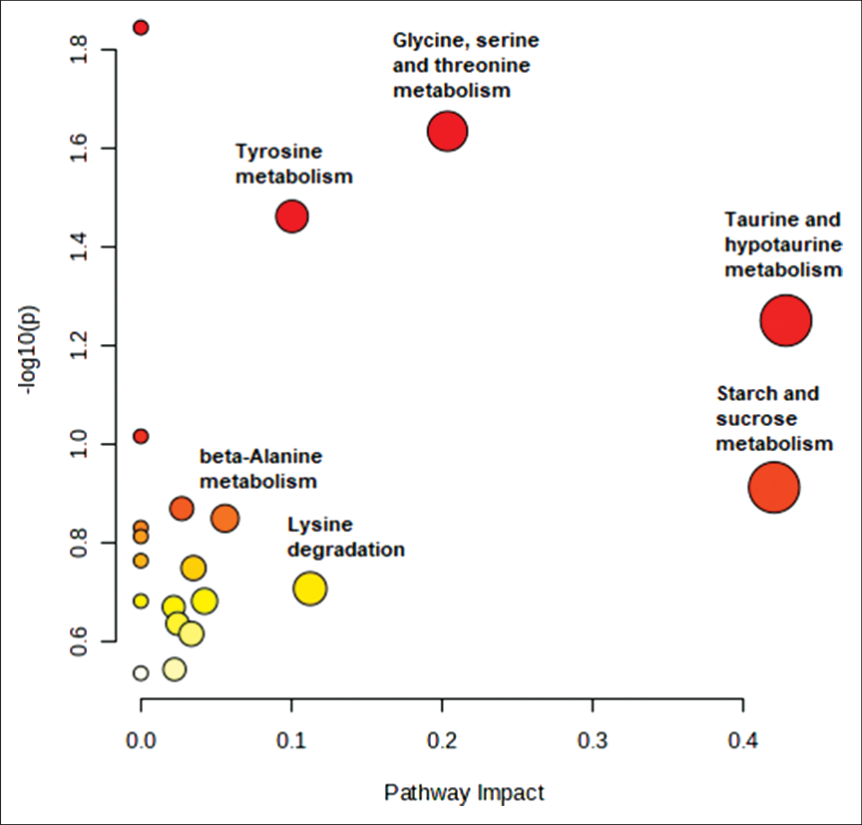
Metabolic pathway analysis involving significant metabolites for the changes. The darker the circle color, the lower p-value and the bigger the circle, the higher the pathway impact.

The role of metabolic pathways in EE is critical for understanding the potential consequences of pain and stress caused by this treatment [35]. Potential biomarkers can provide valuable insights into the effects of EE in bulls by identifying pertinent metabolic and physiological abnormalities. The metabolic pathways and mechanisms involved in EE are important for understanding this procedure’s pain/stress consequences. Potential biomarkers can help extract relevant information about EE and related metabolic and physiological abnormalities. MetPA (University of Alberta, Canada), along with METLIN and KEGG, can generate pathway studies. According to the findings, the taurine and hypotaurine metabolism pathway had the most considerable impact, followed by starch and sucrose metabolism, and the third affected pathway was glycine, serine, and threonine metabolism (Table 3 and Figure 4).

**Table 3:** Metabolic pathways for significantly discriminating metabolites, showing the metabolite hits in each pathway and the pathway impact.

Pathways	Total	Hits	Impact
Neomycin, kanamycin, and gentamicin biosynthesis	2	1	0
Glycine, serine, and threonine metabolism	34	2	0.20373
Tyrosine metabolism	42	2	0.10046
Taurine and hypotaurine metabolism	8	1	0.42857
D-amino acid metabolism	14	1	0
Starch and sucrose metabolism	18	1	0.4207
Pantothenate and CoA biosynthesis	20	1	0.02721
beta-alanine metabolism	21	1	0.05597
Propanoate metabolism	22	1	0
Pyruvate metabolism	23	1	0
Glycolysis/Gluconeogenesis	26	1	0
Galactose metabolism	27	1	0.03499
Lysine degradation	30	1	0.11247
Sphingolipid metabolism	32	1	0
Glyoxylate and dicarboxylate metabolism	32	1	0.04233
Cysteine and methionine metabolism	33	1	0.02184
Arginine and proline metabolism	36	1	0.02442
Pyrimidine metabolism	38	1	0.03356
Primary bile acid biosynthesis	46	1	0.02239
Fatty acid biosynthesis	47	1	0

CoA=Coenzyme A

These results highlight the intricacy of metabolic reactions in bulls experiencing EE and the possible importance of these pathways in understanding the physiological effects of EE. The identification of significant metabolic pathways and plausible biomarkers provides opportunities for more studies emphasizing the reduction of animal pain and stress throughout reproductive processes. These findings can also guide the development of tactics intended to lessen the harmful effects of EE on animal welfare [36, 37].

### Behavioral parameters

A close observation of behavior provides deep insights into an animal’s welfare and physiological status [38]. In bulls, arcing back, struggling, head down and up/down movements, and drooling of saliva are common behavioral responses that indicate an aversion to certain conditions. These behavioral responses were observed in all bulls during EE. These responses subsided immediately after EE was accomplished and were absent at 20 min post-ejaculation. These behavioral responses were associated with hormonal responses and EEG power spectrum variables (Table 4). Similar to the present study, aversive behavior (such as struggling, tendency to lie down, vocalization, and strong muscular movements) was also reported during EE in goats [39] and bulls [40, 41]. The habituation of bulls and rams during EE, due to frequent semen collection, has been reported to decrease the animal’s reaction [42, 43]. Although the bulls used in the present study were habituated to EE, yet they exhibited these behavioral reactions. This behavioral response, associated with increased hormone levels and EEG, indicates pain and stress in bulls in response to EEG.

**Table 4:** Behavioral responses of bulls to EE.

Behavior	Baseline		EE		Relax	
		
	Scale	Frequency	Scale	Frequency	Scale	Frequency
Arc back	–	–	+	100%	–	–
Struggling	–	–	+	100%	–	–
Head down or up	–	–	+	100% (75% down, 25% up and down)	–	–
Drooling	–	–	+	100%	–	–

Scale: –=Absent, +=Present, frequency percentage of bulls showing the behavior. EE=Electroejaculation

The stress response is activated when the central nervous system perceives a threat to homeostasis. This threat elicits a biological response comprising four gene- ral defense systems; behavioral, ANS, neuroendocrine, and immune responses [2]. In the case of many stressors, the first biological response is behavioral; the animal will try to avoid that stressor by escaping from that place [2]. Sometimes, the animal may be in a situation where one particular behavior would not be sufficient to avoid the stressor. For instance, in confinement, such as in this study, where the animal was restrained in a chute, it can manifest signs such as arc back, struggling, head down or up, back and forth movement, drooling of saliva, and kneeling down. In this study, most behavioral signs indicating stress were observed.

The animal’s second line of defense is the ANS, which is based on a “fight or flight” response during stress. The ANS activates various biological systems, including the cardiovascular, gastrointestinal (GIT), exocrine, and adrenal medulla systems, leading to increased vital signs such as heart rate, blood pressure, respiration, and GIT activity. Measuring these vital signs is a challenge in large animals such as bulls. In the same way, the ANS transmits action potential from the stimulus site to the pain centers in the brain through neurons; an instantaneous and the fastest response among all changes initiated in response to stress stimuli. EEG is a real-time graphical representation of tiny (of the microvolt range) spontaneously generated electrical currents of neurons from the cerebral cortex through electrodes placed on various positions on the scalp in humans or heads in other species [5]. The MF and Ptot, along with other frequencies of EEG, have been reported to increase in response to stress and pain in various studies in cattle [26, 32] and goats [27]. The results of this study demonstrate that the EEG indices of pain and stress increased during EE compared with baseline and returned to values similar to baseline values, suggesting that stress and pain respond to EEG.

HPA axis is another mechanism activated in response to stress. This is the primary neuroendocrine mechanism monitored in stress studies and is a crucial system for understanding the effects of stress on animal physiology [2]. Hormones such as cortisol, adrenaline, and noradrenaline are frequently monitored as indicators of stress. In this study, adrenaline and noradrenaline were measured as stress hormones. The results indicate that both hormones were increased during EE compared with baseline, and the values returned to baseline 20 min after EE. This suggests that animals experience stress during EE.

## CONCLUSION

This study provides compelling evidence that EE induces significant stress and pain responses in bulls, as demonstrated by hormonal, EEG, behavioral, and metabolomic assessments. The results indicate that stress-related hormones, including adrenaline, noradrenaline, β-endorphin, and dopamine, significantly increased (p < 0.05) during EE and returned to near-baseline levels 20 min post-ejaculation. EEG analysis revealed a marked increase in MF and Ptot during EE, signifying heightened cortical activity associated with nociception. Behavioral observations further supported these findings, as all bulls exhibited signs of discomfort, including struggling, arched backs, and excessive salivation. Metabolomic analysis identified distinct biochemical changes, with variations in glucose, taurine, norepinephrine, and other metabolites across baseline, stimulation, and recovery phases, highlighting the metabolic stress associated with EE.

This study integrates multiple objective measures – EEG, hormonal profiling, behavioral assessments, and metabolomic analysis – to provide a comprehensive evaluation of pain and stress in bulls undergoing EE. The use of EEG, a real-time neurophysiological tool, adds a novel and reliable dimension to assessing nociception. Furthermore, the metabolomic analysis offers insights into biochemical alterations that may contribute to stress responses, advancing our understanding of the physiological impact of EE beyond traditional welfare assessments.

Despite its strengths, this study has certain limitations. The sample size was relatively small (n = 8 bulls), which may limit the generalizability of the findings. In addition, while EEG and hormonal markers effectively measured acute pain and stress, the study did not evaluate long-term physiological or behavioral effects of repeated EE procedures. The impact of factors such as breed variations, previous exposure to EE, and potential habituation effects was not extensively explored.

Future studies should aim to compare the physiological and behavioral stress responses of bulls across different semen collection methods, such as EE, artificial vagina, and natural mating, to establish the least stressful alternative. In addition, expanding the sample size and including diverse cattle breeds may enhance the robustness of the findings. Longitudinal studies assessing the cumulative impact of repeated EE on reproductive health and overall well-being would also be beneficial.

## AUTHORS’ CONTRIBUTIONS

UK, NYD, GYM, and AM: Conceptualized and designed the study. UK, MWA, KRB, MAB, AAA, and PK, NYD: Conducted experiment and data collection. UK and GYM: EEG data extraction and analysis. AAA and PK, KKH, NYD: Hormonal analysis: UK and AAA: Statistical analyses. PK, UK, and KKH: Drafted the manuscript. UK, PK, KKH, and NYD: Revised the manuscript. All authors have read and approved the final manuscript.
